# Does Antenatal Administration of Magnesium Sulphate Prevent Cerebral Palsy and Mortality in Preterm Infants? A Study Protocol

**DOI:** 10.3934/publichealth.2015.4.727

**Published:** 2015-11-06

**Authors:** Hanne Trap Wolf, Hanne Kristine Hegaard, Anja Bisgaard Pinborg, Lene Drasbek Huusom

**Affiliations:** Hvidovre Hospital, Kettegård Alle 30,2650 Hvidovre, Danmark

## 1. Introduction

The risk of cerebral palsy (CP) is inversely correlated with gestational age at birth [1]. CP is accompanied with life-long consequences for the child, its family and society as a whole. Meta-analyses have indicated that magnesium sulphate may be neuroprotective for the preterm infant, when the drug is given to women at high risk of preterm birth [2],[3]. However, this was recently questioned by a trial sequential analysis (TSA), a statistical method which adjusts for risk of random errors [4]. The TSA demonstrated that additional data are needed before accepting magnesium sulphate as evidence-based therapy for women in preterm labour.

Our aim is to investigate if antenatal magnesium sulphate administrated to women at risk of preterm birth can protect their children against CP.

## 2. Materials and methodology

This trial is ongoing and is performed as a double-blinded, randomized, controlled, multicenter clinical trial. A study population consisting of 500 women, who are at risk of preterm birth at 24 to 32 weeks of gestation, are randomized to receive either intravenous magnesium sulphate or placebo with saline. The women are recruited from 14 obstetrics departments in Denmark. The children are followed up after 18 months of age by a questionnaire (The Ages & Stages Questionnaire), which is a standardized, validated questionnaire containing questions that can reveal signs of CP [5]. The trial is approved by the Scientific Ethics Committee of the Capital Region of Denmark (H-4-2011-024), the Danish Data Protection Agency (HVH-2011-41-6007) and is registered at ClinicalTrials.gov (no. NCT01492608).

Inclusion criteria are: maternal age ≥ 18 years, gestational age 24+0 to 31+6 weeks, singleton or twin pregnancy, preterm rupture of membranes at 24+0 to 31+6 weeks with contractions and expected birth within 2–24 hours, or preterm contractions and expected birth within 2–24 hours and finally anticipated delivery within 2–24 hours of other reasons (for example fetal growth restriction).

Exclusion criteria are major fetal abnormalities, maternal contraindication to magnesium sulphate (e.g. allergy, myasthenia gravis, kidney failure and heart disease), magnesium sulphate administrated for other reasons (e.g. for prevention of eclampsia) and lack of the ability to understand and speak Danish.

### 2.1. Administration of magnesium sulphate

Magnesium sulphate is administrated as a loading dose of five grams infused for 20–30 minutes, followed by a maintenance dose of one gram per hour. Placebo is given in identical appearing doses. The maintenance infusion will be continued until delivery appears, or for 24 hours if delivery does not occur or no longer is considered imminent. The doses that are used in this project are similar to those used in Denmark for prevention of eclampsia. Blood pressure, pulse rate, respiration rate and reflexes are being controlled throughout the period. Also the fetal heart is monitored closely.

### 2.2. Follow-up of the children

The children are followed up after 18 months of age. A questionnaire will be sent to the parents. If signs of CP are revealed from the questionnaire, the children will be examined neurologically by a pediatrician. The effect will be assessed blinded to the treatment.

## 3. Power calculation

A total sample size of 500 patients would allow us to detect or reject a difference in CP of 25% or more with a 5% type 1 error risk. The present trial will with a power of only 13%, not in itself have the power to detect a significant difference between magnesium and placebo treatment. Instead, when the trial is completed, the results will be added to the existing data in a cumulative meta-analysis, in order to ‘close the gap of evidence’ in a TSA, and determine whether magnesium sulphate has an effect. The power of the new meta-analysis will be 63%. We used a one-sided test as a harmful effect of magnesium sulphate on CP seems unlikely according to previous data.

## 4. Current status and future perspectives

To date more than 380 women have been included in the trial. We expect the inclusion period to end in December 2016. Positive results of this trial will support a change of the clinical guidelines concerning the treatment of women with threatening preterm birth.
